# Developmental stage ordering yields greater cranial mineralization sequence resolution than embryo size or days since oviposition: A case study using the gekkotan *Eublepharis macularius* (Blyth, 1854)

**DOI:** 10.1111/joa.70139

**Published:** 2026-03-19

**Authors:** Patrick A. D. Wise, Aaron H. Griffing, Anthony P. Russell

**Affiliations:** ^1^ Department of Biological Sciences University of Calgary Calgary Alberta Canada; ^2^ Department of Chemical and Biological Engineering and Department of Molecular Biology Princeton University Princeton New Jersey USA

**Keywords:** cranial development, developmental chronology, developmental substages, gecko, lizard

## Abstract

Mineralization sequences of cranial elements (often referred to as ossification sequences) are used for a variety of purposes. Believed to be consistent within species (even though they exhibit some variation) and conserved within lineages, they have been assembled using a variety of developmental timetables—absolute time using developmental days; relative time using either increase in size of embryonic dimensions or developmental staging. The relationship between these developmental timetables is unclear in terms of how mineralization sequences are expressed, although they are generally treated as being able to achieve equivalent levels of resolution. Regardless of the developmental timetable employed, mineralization sequences are replete with ties representing simultaneous mineralization of several elements, even though ties are suspected to be rare. Herein we examine the resolution attainable of cranial mineralization events in the leopard gecko by subjecting the same set of embryos, raised under controlled conditions, to sequence analysis using all three of the above‐mentioned timetables, with the working hypotheses being that all three would yield the same level of resolution and that we could improve upon the level of resolution attained for gekkotans so far. We found that developmental stage timetabling yielded far less variability in the determination of mineralization sequence as well as considerably better resolution than those for developmental days or embryonic dimensions. We were able to obtain much greater resolution for the leopard gecko than that so far attained for gekkotans for all three developmental timetables, but especially so when ordering the specimens by developmental stage. Furthermore, we found that subdividing embryonic stages into substages (established using additional morphological features) and assessing intensity of staining of mineralizing elements hold promise for achieving improved levels of resolution. Even so, we were only about 55% successful in resolving the cranial mineralization sequence into a series of unique events, indicating that many such events are so closely spaced in developmental time that apparent simultaneity of element mineralization will be challenging to resolve further.

## Graphical abstract

We employ a set of leopard gecko embryos for which size, day of development, and developmental stage are known to assess which timetable accords with the greatest resolution of cranial mineralization events. Developmental staging yielded the best outcome.

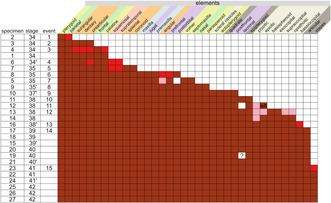

## 1 INTRODUCTION

So‐called ossification sequences (Germain & Laurin, 2009) record the order in which skeletal elements accrue a calcified matrix. Such studies document the first appearance of cartilaginous elements and the first mineralization of bony elements (or of cartilage which later ossifies). The term “ossification” is employed in a nonspecific manner for early stages of skeletogenesis in which the calcification of connective tissue matrix is able to be revealed by particular staining techniques. Skeletogenesis (Hall, 2005) consists of many stages, beginning with aggregations of cells, differentiation of chondro‐osteoblasts in those aggregates, deposition of extracellular matrix (unmineralized osteoid in the case of bones) and then mineralization of that matrix. Due to the lack of histological evidence in studies that rely upon clearing and staining techniques, they can only reveal mineralization events and sequences. Mineralization of matrix precedes true ossification and is the actual event recorded. In the context of this study, therefore, we refer to our observations as mineralization sequences, although what we document is equivalent to the “ossification sequences” described by other authors employing clearing and staining techniques.

Mineralization (“ossification”) sequence data are used to define ontogenetic trajectories of particular species (Bininda‐Emonds et al., 2002; Mitgutsch et al., 2011), to understand the patterning and mechanics of skeletal formation (often applied to the cranial skeleton—Maxwell, 2008b; Schoch, 2004, 2006), as potential characters for systematic study (Laurin et al., 2022; Laurin & Germain, 2011; Maisano, 2002; Maxwell et al., 2010; Monteiro et al., 1999; Shiel et al., 2014) that assist in the understanding of morphological evolution (Fröbisch et al., 2010; Maxwell, 2008b; Smith, 2002; Wiesbecker & Mitgutsch, 2010), and in studies relating to developmental modularity (Chapelle et al., 2020; Goswami et al., 2009; Laurin, 2014).

Despite being an important attribute of complex, multi‐tissue structures (Cooper et al., 2025; Griffing et al., 2022; Santos‐Durán et al., 2025), the processes determining mineralization sequences are not well understood (Maxwell, 2008a). Factors influencing them may be intrinsic to the embryo itself, such as embryonic movement, morphological modularity, chondrification sequences, phylogenetic inheritance, functional constraints (Adriaens & Verraes, 1998; Asakura & Kawabe, 2022; Harrington et al., 2013; Spiekman & Werneburg, 2017; Wagemans & Vandewalle, 2001) or adaptations to embryonic life (Maxwell, 2008a). The latter are more likely for free‐living embryos, such as fishes (Marinho, 2023) and amphibians (De Sa, 1988) than they are for amniotes (with the possible exception of marsupials; Goswami et al., 2009, which are extremely altricial and express very little skeletal mineralization at birth, these levels being comparable to those of eutherian embryos even though they are technically not embryos after birth). Other determining factors may be extrinsic, and therefore easier to examine in a laboratory setting, such as the geographic source of material (thereby capturing natural variation) and the influence of temperature, humidity (Maxwell, 2008a), or light (Zhang et al., 2016).

Mineralization (“ossification”) sequences have been considered by some to be stereotyped (Maxwell, 2008a; Maxwell et al., 2010), although they are not invariant (Maxwell & Harrison, 2008) and may exhibit intraspecific variation (Maxwell et al., 2010; Shiel & Greenbaum, 2005). Such heterochronic variation (Laurin et al., 2022) has been reported to vary from minor (Maxwell, 2008a) to extensive (Hanken & Hall, 1984; Shiel et al., 2014; Spiekman & Werneburg, 2017), even between populations of the same species (Moore & Townsend Jr., 2003). Intraspecific variation is problematic because it introduces sequence ambiguity (Wiesbecker & Mitgutsch, 2010) and thus requires consideration (Garn & Rohmann, 1960; Laurin et al., 2022). Regardless of the degree of variation in such sequences, it is none‐the‐less believed that there is deep‐time conservation of the basic characteristics of mineralization sequences among squamate reptiles (Chapelle et al., 2020; Hugi et al., 2010; Khannoon & Evans, 2020; Skawiński et al., 2023).

The underlying assumption that allows mineralization sequences to be employed in such versatile ways (notwithstanding their potential inherent variability) is that they are regarded as being consistent within species (Mohammed, 1988) and directly comparable between species, with problems of comparative staging and standardization being eliminated because the sequence itself is deemed to be the criterion of standardization (Smith, 2002). Various methods are available for comparing sequence data (including data compiled using different chronologically related ordering processes; Harrington et al., 2013; Laurin et al., 2022; Sánchez‐Villagra et al., 2008; Shiel et al., 2014; Wiesbecker & Mitgutsch, 2010), such as parsimony analysis, heterochrony plots, event‐pairing (Bininda‐Emonds et al., 2002; Hugi et al., 2010; Maxwell, 2008a; Sánchez‐Villagra et al., 2008; Wiesbecker & Mitgutsch, 2010), and rank ordering of appearance (Harrington et al., 2013; Wiesbecker & Mitgutsch, 2010). Such approaches relate the mineralization sequence datum for each element to every other element, but ultimately must rely upon the degree of resolution that can be gained from the sample of embryos available for study. Except for species that are routinely raised in laboratory situations, adequate sampling may prove challenging. Even for species whose development is well understood, the relationship between various developmental timetables (absolute time; body size; developmental stage) and mineralization sequences remains unclear.

Investigations of mineralization sequences reveal that although sequential mineralization of individual elements features prominently, there is a high incidence of apparent simultaneity, resulting in many ties (Harrington et al., 2013; Hugi et al., 2010, 2012; Khannoon & Evans, 2020; Laurin et al., 2022; Monteiro et al., 1999; Moore & Townsend Jr., 2003; Ollonen et al., 2018; Skawiński et al., 2023). True simultaneity has, however, been advocated to be rare (Bininda‐Emonds et al., 2002), with ties instead resulting from lack of temporal resolution. It has been assumed that mineralization of two or more elements commencing at exactly the same time is unlikely (Hugi et al., 2010), although it is probable that those under similar hormonal control might be expected to become evident within a very short developmental time frame. In a practical sense, therefore, ties may relate to initiations that are consistently so close in time that they seemingly defy segregation (Wiesbecker & Mitgutsch, 2010).

If such simultaneous onset of mineralization is actually less common than implied by accounts in the literature, it is possible that lack of resolution is a function of sampling periodicity employed and/or inaccurate representation of developmental time in relation to the specimens being examined. For lizards, several of which have been the subject of mineralization sequence analysis (Abdala et al., 1997; Good, 1995; Hernández‐Jaimes et al., 2012; Hugi et al., 2010, 2012; Jerez et al., 2015; Khannoon & Evans, 2020; Maisano, 2001, 2002; Monteiro et al., 1999; Ollonen et al., 2018; Rieppel, 1992, 1993, 1994; Roscito & Rodrigues, 2012a; Skawiński et al., 2023; Werneburg et al., 2015), problems of resolution are evident (Table 1). The number of events reported to cover the commencement of mineralization of cranial elements ranges from two to eight (Table 1). All such accounts incorporate one or more events in which mineralization of 10 or more elements apparently commences simultaneously (Table 1), thus accounting for between 34.4% and 75.0% of commencement of cranial element mineralization recorded for that species.

**TABLE 1:** Summary of cranial mineralization sequence information pertinent to lizards as extracted from the literature.

Species	Event							
	1	2	3	4	5	6	7	8
*Lacerta agilis* (28)	St 34; **5**	St 35; **20**	St 37; **3**					
*Liolaemus quilmes* (31)	14 dpo; **3**	20 dpo; **17**	27 dpo; **9**	36 dpo; **2**				
*Amphisbaena darwini* (23)	St IIIa **16**	St IIIc **6**	St IIId **1**					
*Liopholis whitii* (28)	15.7 mm **1**	18.6 mm **9**	22.0 mm **10**	26.5 mm **8**				
*Lerista bougainvillii* (28)	16.8 mm **21**	20.3 mm **7**						
*Hemiergis peronii* (29)	12.4 mm **19**	17.0 mm **3**	18.1 mm **7**					
*Saiphos equalis* (28)	17.1 mm **5**	18.8 mm **8**	23.4 mm **13**	26.0 mm **2**				
*Alopoglossus bicolor* (23)	St 35 **4**	St 39 **17**	St 39–40 **2**					
*Calyptommatus sinebrachiatus* and *Nothobachia ablephara* (32)	16 dpo **1**	20–21 dpo **1**	20–21 dpo **1**	20–21 dpo **9**	20–21 dpo **1**	26 dpo **8**	Prehatching stages **11**	
*Varanus panoptes* (35)	39 mm **3**	41 mm **1**	50 mm **18**	62 mm **4**	70 mm **2**	81 mm **2**	82 mm **4**	102 mm **1**
*Mabuya* sp. (26)	St 32–33 **1**	St 34 **7**	St 39 **16**	St 40 **2**				
*Pogona vitticeps* (31)	18 dpo **1**	24 dpo **7**	28 dpo **14**	32 dpo **2**	36 dpo **5**	40 dpo **1**	48 dpo **1**	
*Tarentola annularis* (30)	Late St 34 **19**	Late St 35 **11**						
*Lepidodactylus lugubris* (29)	St 34 **3**	St 35 **4**	St 36 **4**	St 37–38 **10**	St 40 **8**			
*Heremites auratus* (21)	St 34 **2**	St 37 **18**	St 38 **1**					

*Note*: The number of skeletal elements included in each account is indicated in parentheses after the species name in the first column. The number of events identified in each published mineralization sequence is indicated by the number of column entries occurring in the Events columns (maximum of eight entries). The primary temporal organizing criterion for each species is indicated (St, developmental stage; dpo, days post‐oviposition; xmm, size [HL and/or SVL] dimension in millimeters). For some species more than one temporal organizing criterion is provided—supplementary criteria are identified by superscript symbols, as follows for each pertinent publication: ^+^ dpo; ^#^ SES; ^^^ Size; * Stage. The number in bold following the primary organizing criterion in each cell indicates the number of cranial bones initiating mineralization in that event. Cells with gray background shading indicate mineralization initiation events in which 10 or more elements are tied. Cells with pink backgrounds indicate initiation events for which there are no ties. The species examined and family, and the source of the information about mineralization sequences are as follows (listed in chronological order of publication): *Lacerta agilis* Linnaeus, 1758 Lacertidae (Rieppel, 1994 ^^^); *Liolaemus quilmes* Etheridge, 1993 Liolaemidae (Abdala et al., 1997*; Khannoon & Evans, 2020); *Amphisbaena darwini* Duméril and Bibron, 1839 Amphisbaenidae (Monteiro et al., 1999). Idiosyncratic staging scheme; *Liopholis whitii* (Lacépède, 1804) Scincidae (Hugi et al., 2010, 2012); *Lerista bougainvillii* (Gray, 1839) Scincidae (Hugi et al., 2012); *Hemiergis peronii* (Gray, 1831) Scincidae (Hugi et al., 2012); *Saiphos equalis* (Gray, 1825) Scincidae (Hugi et al., 2012); *Alopoglossus bicolor* (Werner, 1916) Alopoglossidae (Hernández‐Jaimes et al., 2012); *Calyptommatus sinebrachiatus* (Rodrigues, 1991) Gymnophthalmidae and *Nothobachia ablephara* (Rodrigues, 1984) Gymnophthalmidae (Roscito & Rodrigues, 2012a, 2012b); *Varanus panoptes* (Storr, 1980) Varanidae (Werneburg et al., 2015 ^+^#^); *Mabuya* sp. Scincidae (Jerez et al., 2015); *Pogona vitticeps* (Ahl, 1926) Agamidae (Ollonen et al., 2018 ^#^*); *Tarentola annularis* (Geoffroy Sainte‐Hilaire, 1827) Phyllodactylidae (Khannoon & Evans, 2020 ^+#^); *Lepidodactylus lugubris* (Duméril and Bibron, 1836) Gekkonidae (Skawiński et al., 2023 ^+^); *Heremites auratus* (Linnaeus, 1758) Scincidae (Candan et al., 2024).

The resolution of such sequences influences the interpretation of the data (Maxwell & Harrison, 2008) and their application to various questions of biological import. Investigation of mineralization sequences has often been based upon a relatively small number of developmental stages and/or relatively few specimens (for example, Abdala et al., 1997; Hernández‐Jaimes et al., 2012; Hugi et al., 2010, 2012; Jerez et al., 2015; Khannoon & Evans, 2020; Monteiro et al., 1999), often with conspicuous gaps in the assembled developmental series (Table 1).

Over the past 30 years (Table 1) there appears to have been little improvement in the degree of resolution of events in the cranial mineralization sequence data available for lizards, despite the employment of new investigative techniques (Ollonen et al., 2018). To date, the most complete resolution for lizard cranial elements identifies eight events that collectively account for 35 elements (Werneburg et al., 2015; Table 1). For geckos, the most completely resolved sequence identifies five events accounting for 29 elements (Skawiński et al., 2023, see Table 1).

In the absence of longitudinal data, many specimens will be required for full resolution of cranial mineralization sequences to be attained, especially for highly condensed portions of the emerging pattern (Chapelle et al., 2020; Maxwell, 2008b). Despite the fundamental assumption that the sequence itself is the criterion of standardization, a strategy of sampling that can resolve the sequence to its greatest extent is required (Laurin et al., 2022), because the onset of mineralization of individual elements is the criterion upon which the sequence is established.

Three chronologically based approaches underpinning the investigation of cranial mineralization sequences have been employed. One, especially useful for lineages in which similar features may be expressed differentially in relation to each other, is developmental staging (Blanc, 1974; Dhouailly & Saxod, 1974; Dufaure & Hubert, 1961; El Mouden et al., 2000; Griffing et al., 2019; Lemus, 1967; Lemus et al., 1981; Milaire, 1957; Muthukkarruppan et al., 1970; Noro et al., 2009; Pasteels, 1956; Peter, 1904; Rieppel, 1994; Sanger et al., 2008; Thapliyal et al., 1973; Wise et al., 2009). This employs recognizable markers that exhibit a conserved and reliable sequence of appearance through developmental time within a given species, regardless of environmental vagaries that may alter the real‐time rate of development and/or the size of embryos (Sinervo et al., 1992). The criteria for recognizing developmental stages may not, in themselves, be important for understanding particular developmental phenomena but, because they occur in a consistent and unvarying sequence, can be used to set a relative timetable against which other, associated events can be measured. Several studies investigating mineralization sequences within lizard head skeletons have used developmental staging as the primary chronological reference scale (Candan et al., 2024; Hernández‐Jaimes et al., 2012; Jerez et al., 2015; Khannoon & Evans, 2020; Monteiro et al., 1999 [using an idiosyncratic staging scheme]; Skawiński et al., 2023; Rieppel, 1994; Table 1). More recently developmental staging has been expressed with reference to a modified stage‐based scheme, the Standard Event System (SES) (Ollonen et al., 2018; Polachowski & Werneburg, 2013; Werneburg, 2009; Werneburg et al., 2015), which allows external morphological features of embryos of different species to be aligned using the same numerical designator for each SES stage.

An alternative means of recording the passage of time during embryonic development is the employment of day of development (Abdala et al., 1997; Roscito & Rodrigues, 2012a; Ollonen et al., 2018; Table 1), recorded as days post‐oviposition (dpo). This employs real, rather than relative, time and has also been employed as the primary ordering criterion in the documentation of lacertilian mineralization sequences. The number of days taken to eclosion may, however, be quite variable (Deeming & Ferguson, 1991), depending upon lineage‐specific patterns and/or environmental conditions (e.g., Andrews et al., 2013; Andrews & Mathies, 2000).

A third approach to investigating the chronology of cranial mineralization events has employed size as the primary surrogate representing the passage of developmental time (Hugi et al., 2010, 2012; Werneburg et al., 2015; Table 1). The relationship between developmental differentiation and size is, however, poorly understood (other than larger generally being assumed to be older). To sample appropriately using size as a criterion, it is necessary to insert gaps into the size‐based sampling range, which may be especially problematic if mineralization events are closely spaced (but not simultaneous) in developmental time.

Some authors have employed more than one of these representations of the passage of developmental time concurrently (Abdala et al., 1997; Khannoon & Evans, 2020; Ollonen et al., 2018; Rieppel, 1994; Skawiński et al., 2023; Werneburg et al., 2015; Table 1), but in all cases one “clock” has served as the primary criterion for ordering the presentation of results.

We herein explore the cranial mineralization sequence for the eublepharid leopard gecko (*Eublepharis macularius*). We trace the initiation of mineralization of all 35 of the cranial elements described for this species by Wise (2015) plus the scleral ossicles, which Wise (2015) did not incorporate in his study. We evaluate the sequence of mineralization against all three developmental timing approaches mentioned above. Our objectives are to determine which approach, if any, comes closest to complete resolution. In addition, we seek to improve the currently meager resolution of pattern available for gekkotans (Khannoon & Evans, 2020; Skawiński et al., 2023; Table 1).

We employ a relatively densely sampled developmental series of staged embryos (Wise et al., 2009) of *Eublepharis macularius* that captures the embryonic period incorporating all cranial mineralization initiation events. The same series of embryos provides the data for the application of each of the chronological timetables (embryo size, day of development, and developmental stage). Our working hypotheses are as follows. (1) Based upon (unstated) assumptions in the literature, all three approaches will result in the same outcome. (2) With regard to cranial mineralization sequence for gekkotans, a greater level of resolution will be able to be attained than is currently available (Khannoon & Evans, 2020; Skawiński et al., 2023; Table 1), with resolution being attained to at least the level of the best‐resolved sequences for other lizards (Table 1).

## 2 MATERIALS AND METHODS

Leopard gecko (*Eublepharis macularius*) eggs were incubated at 28 ± 1°C (the female‐biased producing temperature; Bull, 1987; Viets et al., 1993, 1994) to reduce any variance that may be due to sex. Embryos were sacrificed in accordance with Canadian Council on Animal Care guidelines (all necessary approvals for this study were obtained via University of Calgary Animal Care protocol BI2006‐37). At the time of sacrifice eggs were cooled to 4°C to reduce the metabolic rate and responsiveness of the embryos (American Society of Ichthyologists and Herpetologists, 2004; Canadian Council on Animal Care, 2010) and a 1 mm incision made in the underside of the egg, through which 10% neutral buffered formalin was injected. Whole eggs were then fixed in 10% neutral buffered formalin for 48 h at room temperature (RT). Following rinsing in running tap water for 24 h, embryos were removed from their eggs and stored in 70% ethanol (at RT). The embryos investigated in this study form part of the larger developmental series employed in the establishment of the developmental staging data for the leopard gecko (Wise et al., 2009). All embryos are thus of known developmental stage and day of incubation. For captive bred and raised *E. macularius*, oviposition occurs at Stages 28–29 and hatching at Stage 42 (as documented by Wise et al., 2009), although Griffing et al. (2019) recognized Stage 43 in geckos, characterized by recession of the hemipenes and their female homologs. Because the embryos were exclusively female, we were able to avoid variation attributable to sexual differences.

Forty‐five embryos were found to be pertinent to this study, covering developmental Stages 34–41 (Wise et al., 2009), the total span of developmental stages revealed to incorporate the initiation of mineralization of all cranial elements (see below). Examination of the total *in ovo* developmental sequence revealed that no cranial skeletal elements are evident prior to stage 34 and all cranial elements have made an appearance by Stage 41. These eight developmental stages (of the 15 in total identified from egg laying to hatching—Wise et al., 2009), encompass 21 days of the 52+/−2 developmental days between laying and hatching (when raised under the incubation conditions described above).

To examine the sequence of mineralization of cranial elements in relation to size of the embryos, we used head length as the measurement criterion. We chose this parameter rather than snout‐vent length (SVL) or total length (TL) of the embryo for two reasons. First, cranial mineralization is likely to be more closely related to head size than overall body size. Progression of development occurs along the craniocaudal axis, so relative proportions of head and body will change with time. Head dimensions are therefore likely the most pertinent in relation to cranial mineralization sequences and progression. Second, overall body length is difficult to measure accurately in early developmental stages due to the curled nature of the embryos. Head length was employed by Rieppel (1994) in his assessment of cranial mineralization sequencing for *Lacerta agilis* Linnaeus, 1758 (Table 1), and Griffing et al. (2018) in their study of postnatal skull development in sphaerodactylid gekkotans, but other authors using a length dimension as a reference marker for progress of cranial mineralization have employed either SVL (Werneburg et al., 2015), TL (Hugi et al., 2010, 2012), or both (Ollonen et al., 2018). The latter authors made various measurements of head dimensions of embryos of *Pogona vitticeps* (Ahl, 1926) but did not directly employ them in the establishment of their staging criteria or in consideration of cranial mineralization sequence. Head length was measured from the tip of the upper jaw along a line following the upper jaw margin (in lateral view) to a point at which the line intersects the dorsum. This approach permitted a consistent way of measuring head length that was not subject to the potential vagaries introduced by cranial flexure or the dorsal bulging of the brain early in development. HL was measured three times for each individual by one of us (PADW) and its average value calculated. With reference to day of development (dpo), embryos were harvested at known intervals after laying (Table 2).

**TABLE 2:** Alternative developmental timetables employed in determining and resolving cranial mineralization sequencing in the leopard gecko (*Eublepharis macularius*).

Specimen number (see Figures 1, 2, 3)	Head length (HL) in mm (see Figure 1)	Day of development (dpo; see Figure 2)	Developmental stage (see Figure 3)	Developmental stage and substage (see Figure 3)
2	7.5	16	34	34
3	7.6	21	34	34
4	7.8	22	34	34
1	7.0	21	34	34
6	8.3	20	34	34′
7	8.6	23	35	35
8	9.0	26	35	35
5	8.0	21	35	35
9	9.0	24	35	35′
10	10.8	25	37	37′
11	11.0	23	38	38
12	11.2	26	38	38
13	12.6	28	38	38
14	12.8	31	38	38
16	13.5	30	38	38’
17	13.6	33	39	39
18	14.3	34	39	39
15	13.4	35	39	39’
20	16.5	36	40	40
19	16.2	38	40	40
21	16.8	42	40	40’
23	18.7	36	41	41
22	19.1	41	41	41
24	17.2	45	41	41’
25	19.3	44	42	42
26	19.3	49	42	42
27	22.4	50	42	42

*Note*: Specimen number (left column) for each of the 27 representative specimens corresponds to that for which mineralization sequence data are presented in Figures 1, 2, 3, and is presented here in the sequence encountered in Figure 3 (see below). Head length is listed in column 2. Size is cross‐referenced to the continuous sequences, day of development (dpo), and developmental stage, in columns 3–5 that are arranged using real (dpo) or relative (developmental stage) time data. Developmental stage (column 4) for the 27 representative embryos for depicting mineralization sequence data (Figures 1, 2, 3) is used as the primary ordering criterion against which the other timetables are compared (see text for reasons). Columns 3–5 report the time‐based developmental status of each of the 27 representative embryos.

Staging was established for a series of 111 embryos, using externally visible criteria (Wise et al., 2009). Of these, 45 of the 54 embryos encompassing developmental Stages 34–41 (Wise et al., 2009, Table 3) were used in the current study. For each of these, the developmental day was known. Because of the lengthy duration of many of the developmental stages, we further subdivided them by documenting additional morphological changes, not associated with classical staging criteria, that occur later within the confines of a given stage, prior to entry into the next developmental stage. We thus established substages, each recognizable by one or more morphological criteria, thereby establishing a later phase of development within that numbered stage (and not as a bridge between recognized stages). Assignment to substage was helpful for clarifying the sequence of mineralization because the longer the temporal duration of a particular stage, the greater the likelihood that several mineralization initiations will occur within that developmental stage. Other authors, when exploring cranial mineralization sequences, have alluded to compartmentalization of development by subdividing stages (Werneburg et al., 2015—SES stages subdivided by ascending SVL and dpo; Ollonen et al., 2018—ascending dpo within the same SES stage; Khannoon & Evans, 2020; Skawiński et al., 2023—developmental stage subdivided into early, mid, and late sectors; Table 1). No morphologically based recognition criteria, other than increasing size, associated with such ostensible subdivision have, however, been described in such studies.

**TABLE 3:** Comparison of resolution of cranial mineralization sequences for gekkotans.

*Tarentola annularis* (Khannoon & Evans, 2020, Table 1)	*Lepidodactylus lugubris* (Skawiński et al., 2023, Table 2)	*Eublepharis macularius* (this study, Figure 3)	*Eublepharis macularius* (this study, Figure 3, segregation by intensity of staining)
**St 34**	**St 34**	**St 34**	**St 34**
Basisphenoid, Dentary, Ectopterygoid, Epipterygoid, Frontal, Jugal, Maxilla, Nasal, Parietal, Palatine, Pterygoid, Postorbitofrontal, Prearticular, Prefrontal, Premaxilla, Quadrate, Septomaxilla, Surangular, Vomer	Prearticular, Pterygoid, Surangular	Parietal, Pterygoid	Pterygoid
		**St 34** Dentary, Prearticular, Surangular	**St 34** Parietal
		**St 34** Frontal, Palatine	**St 34** Dentary, Prearticular, Surangular
			**St 34** Frontal
			**St 34** Palatine
		**St 34’** Squamosal, Supratemporal	**St 34’** Squamosal, Supratemporal
**St 35**	**St 35**	**St 35**	**St 35**
Articular, Basioccipital, Coronoid, Exoccipital, Opisthotic, Parasphenoid, Prootic, Splenial, Squamosal, Stapes, Supraoccipital	Dentary, Frontal, Parietal, Squamosal	Coronoid, Splenial	Coronoid, Splenial
		**St 35** Angular, Jugal, Maxilla, Postorbital, Prefrontal, Premaxilla	**St 35** Jugal, Maxilla, Postorbital, Premaxilla
		**St 35** Septomaxilla, Vomer	**St 35** Angular, Prefrontal
			**St 35** Septomaxilla, Vomer
			**St 35’** Nasal, Parasphenoid
	**St 36** Maxilla, Nasal, Prefrontal, Vomer	**St 35’** Nasal, Parasphenoid	
	**St 37–38** Coronoid, Epipterygoid, Exoccipital, Jugal, Palatine, Premaxilla, Postorbitofrontal, Quadrate, Septomaxilla, Splenial	**St 37’** Ectopterygoid, Prefrontal, Quadrate, Scleral ossicles	**St 37’** Ectopterygoid, Prefrontal, Quadrate, Scleral ossicles
		**St 38** Epipterygoid, Lacrimal	**St 38** Epipterygoid, Lacrimal
		**St 38** Epiotic, Prootic	**St 38** Epiotic
		**St 38** Basisphenoid, Exoccipital, Opisthotic, Supraoccipital	**St 38** Prootic
			**St 38** Basisphenoid, Exoccipital
			**St 38** Opisthotic, Supraoccipital
		**St 38’** Basioccipital	**St 38’** Basioccipital
		**St 39** Articular	**St 39** Articular
	**St 40** Articular, Basioccipital, Basisphenoid, Ectopterygoid, Opisthotic, Parasphenoid, Prootic, Supraoccipital	**St 40** Stapes	**St 40** Stapes

*Note*: For *Tarentola annularis* (Column 1. Khannoon & Evans, 2020) only two mineralization initiation events, accounting for 30 cranial elements, were documented (see Table 1). For *Lepidodactylus lugubris* (Column 2. Skawiński et al., 2023) five mineralization initiation events were identified for 29 cranial elements (see Table 1). In this study, we identified 15 mineralization events (Column 3) for 36 cranial elements of *Eublepharis macularius* using the first appearance of Alizarin staining, regardless of staining intensity. Employing intensity of staining on first appearance, we were able to refine this to 20 events for 36 elements for *Eublepharis macularius* (Column 4). Cells with gray background shading indicate mineralization initiation events in which 10 or more elements are tied. Cells with pink backgrounds indicate initiation events for which there are no ties.

We thus employ two versions of the *Eublepharis macularius* staging table—that set forth by Wise et al. (2009), and a more finely subdivided version incorporating the later‐occurring substages mentioned above (Table 2). We were able to subdivide Stages 34, 35, 37, 38, 39, 40 and 41 into smaller subunits. Wise et al. (2009) describe the features demarcating the commencement of each developmental stage. We here outline the features by which substages of the seven developmental stages listed above are recognized. These substages are identified by the “prime” symbol (for example, 34′ signifies the later recognized substage within Stage 34).

Stage 34′

Eye: Developing upper and lower eyelids project from the circumferential perimeter of the eye as a thin ribbon‐like sheet; that portion of the eye exposed between developing eyelids is oval in shape.
Branchial arches: Lower jaw equal in length to craniofacial region.

Stage 35′

Cranial: Tympanum formed.

Stage 37′

Eye: Thickening of ocular margin of both upper and lower eyelids spreads to caudal margin of eye.
Branchial arches: Upper and lower jaws of similar length.
*Limbs*: Claws present on all digits.

Stage 38′

Cranial: Swelling in the region of the presumptive parietal bone begins to merge with the contour of the head and becomes less distinct.
Scales: Scales on the head spreading from ventral to dorsal; labial scales faintly visible on lower jaw; forelimbs completely scaled.

Stage 39′

Scales: Labial scales distinct on both lower and upper jaw.

Stage 40′

Pigmentation: Juvenile banded pigmentation pattern begins to develop; forelimb pigmentation is weakly visible.

Stage 41′

Scales: Tubercular scales keeled on neck but remain flat on head.
*Pigmentation*: Juvenile pigmentation pattern achieved.
*Overall*: Embryo is 75 to 100% hatchling length.

Following measurement of HL (and TL) of the embryos, and determination of stage (Wise et al., 2009; Table 2) and substage (see above and Table 2), embryos were cleared and stained to enable visualization of skeletal mineralization. Double staining of embryos was accomplished in the following fashion. (1) Formalin fixation, (2) two 24 h rinses in distilled water; (3) staining in aqueous Alcian blue for 24 h; (4) 6 h in 100% ethanol; (5) 1 h rinses in each of 95%, 70%, 40%, 15% ethanol; (6) 2–4 h in distilled water; (7) trypsin digestion at 38°C for a variable amount of time depending upon the overall size the embryo; (8) 24 h in distilled water; (9) 0.02% aqueous Alizarin red for 2 h; (10) 24 h in each of 0.5% KOH/glycerine in the following concentrations—3:1; 1:1; 1:3; (11) storage in glycerin (with thymol crystals).

Double‐stained embryos were used, in combination with single‐stained embryos, to distinguish dermal bones from chondrocranial and splanchnocranial elements, which have a cartilaginous precursor. Our staining methodology was adapted from that of Filipski and Wilson (1985) by omitting the Sudan Black B step. Thirteen embryos were double‐stained. Thirty‐two embryos were single‐stained using Alizarin red only, by omitting Steps 2 and 3, above. The 32 single‐stained embryos were used to establish the mineralization sequence, all of which took up stain in the endolymphatic system, which served as a control for the effectiveness of the stain and indicated that staining was possible, even if no skeletal elements could be visualized. In total, 27 embryos (Table 1) were sufficient for the establishment of as fully resolved as possible mineralization sequences employing all three developmental timetables. In an attempt to further resolve the sequence of mineralization, we also recorded the intensity of staining of elements (weak, moderate or strong) for any embryo for which this level of discrimination was possible. We recognize that weaker staining intensity might reflect other factors, such as the overall size of the developing primordium, rather than distinct developmental timing, so we consider this phenomenon only as a tentative supplementary factor that requires further investigation.

Recently, it has been noted that typical clearing and double‐staining procedures (using Alizarin red S and Alcian Blue), such as that employed for some of the specimens in this study, are conducted under acidic conditions, which may inhibit uptake of Alizarin red S (Walker & Kimmel, 2007; Zinck & Franz‐Odendaal, 2021). Hautier et al. (2014) note that typical clearing and staining procedures are adequate for the evaluation of mineralization sequences at the scale of the entire skeleton, although they might be less sensitive to minute mineralization centers in parts of elements, which are more readily detected using CT scans. Furthermore, Werneburg et al. (2015) note that the employment of Alizarin red S alone avoids the influence of the acidic medium in the staining procedure and is thus more accurate for determining the onset of mineralization, although ineffective for the observation of chondrification. Our employment of 32 embryos cleared and stained with Alizarin red S only, plus a further 13 double‐stained embryos, provided us with the opportunity to combine the two types of data to more effectively document the cranial mineralization sequence in *Eublepharis macularius*. Notwithstanding this, however, employment of acid‐free staining techniques (Walker & Kimmel, 2007) in the future may render more nuanced results (Skawiński et al., 2023).

## 3 RESULTS

### 3.1 Resolution of mineralization using size (head length—HL) as the proxy for developmental time

**FIGURE 1:**
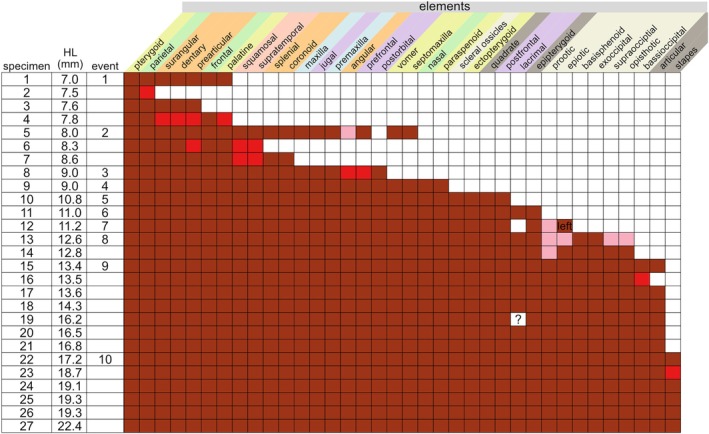
Cranial element mineralization sequence data gleaned from 27 embryos of *Eublepharis macularius* with the specimens (1–27) arranged in size (head length—HL) order (see Table 2). The “event” column identifies the 10 mineralization events documented in the text. Filled cells record evidence of mineralization as revealed by staining with Alizarin red, with pink fill indicating weak staining, light red fill indicating moderate staining, and dark red fill indicating strong staining. Cranial entities are listed across the top of the figure, with color‐coded background fill indicating groups of purportedly anatomically associated elements (Evans, 2008; Schoch, 2006), as follows: Palatal elements yellow; skull roofing bones light green; lower jaw bones coral; temporal bones pink; tooth‐bearing bones of the upper jaw light blue; circumorbitals purple; chondrocranial elements (and scleral ossicles) clear; splanchnocranial elements gray.

Ordering the embryos in an increasing sequence of size has been employed as a surrogate for developmental time in relation to mineralization sequences of lizards (Hugi et al., 2010, 2012; Werneburg et al., 2015; see Table 1). The greatest resolution obtained for *Eublepharis macularius* using this approach resulted in the identification of 10 distinct events, indicated by the steps documenting the addition of newly mineralized elements reading from top left to bottom right of Figure 1. It is evident that early in the developmental process, the correlation between HL and mineralization sequence is quite weak (Figure 1), and variability between embryos is quite high. These data reveal considerable apparent simultaneity of initiation of mineralization of cranial elements.

The 10 mineralization events revealed by ordering the embryos by HL are as follows (Figure 1): (1) pterygoid, parietal, surangular, dentary, prearticular, frontal, palatine; (2) squamosal, supratemporal, splenial, coronoid, maxilla, jugal, premaxilla, angular, prefrontal, vomer, septomaxilla; (3) postorbital; (4) nasal, parasphenoid; (5) scleral ossicles, ectopterygoid, quadrate, postfrontal; (6) lacrimal, epipterygoid; (7) prootic, epiotic; (8) basisphenoid, exoccipital, supraoccipital, opisthotic; (9) basioccipital, articular; (10) stapes. Some embryos of smaller size exhibit more extensive initiation of mineralization than do some larger ones, generating a jagged edge on the mineralization front that advances across (Figure 1). From two to 11 elements are inferred to initiate mineralization simultaneously, and no further resolution can be obtained for these. Only two elements are recognized as initiating mineralization alone (Figure 1).

Given the density of sampling (permitting observation of several embryos at similar points along the developmental trajectory) we tentatively suggest that weak staining represents an early phase of mineralization, followed by moderate and then strong staining. Staining intensity offers the potential for breaking ties for seemingly simultaneous events. For example, for the embryo of HL 8.0 mm (specimen 5, Figure 1) the angular stains weakly, indicating that its initiation of mineralization occurred later than that of the other elements identified as being mineralized in this specimen. Interpreted in this way, the following additional events can be advocated (Figure 1): the parietal initiates mineralization later than the other elements identified as mineralizing in event (1) (above); mineralization of the surangular, dentary, prearticular and palatine occurs later than the pterygoid and parietal; mineralization of the squamosal, supratemporal, angular and prefrontal occurs later than mineralization of the other elements identified in event (2); and mineralization of the prootic, epiotic, supraoccipital and opisthotic occurs later than mineralization of the other elements identified in event (7). If these additional pieces of evidence are factored in, then 14 mineralization events, rather than the 10 outlined above (Figure 1), are identifiable.

### 3.2 Resolution of mineralization sequence using developmental day (dpo) as the proxy for developmental time

Recently, there has been a trend to documenting the development of lizards employing developmental days, and this timetable has been employed as the primary chronological ordering principle against which mineralization sequences are established (Abdala et al., 1997; Ollonen et al., 2018; see Table 1). Such an approach may provide a finer‐scaled subdivision of the developmental program, but different embryos raised under the same conditions can exhibit different rates of development over the same time period (Table 2), possibly due to the developmental stage attained at the time of oviposition. This potentiality could be assessed by carefully recording the clutches to which particular eggs belong and testing for differences between clutches (something in hindsight we omitted to do). Thus, although the developmental sequence may be able to be more finely subdivided by employing the day of development criterion, and given that this approach may simplify the harvesting of eggs for embryos (because day of development can be determined a priori, whereas HL or stage of development can only be determined after harvesting), it does not necessarily follow that this sampling criterion will bring greater resolution to the documentation of mineralization sequences.

**FIGURE 2:**
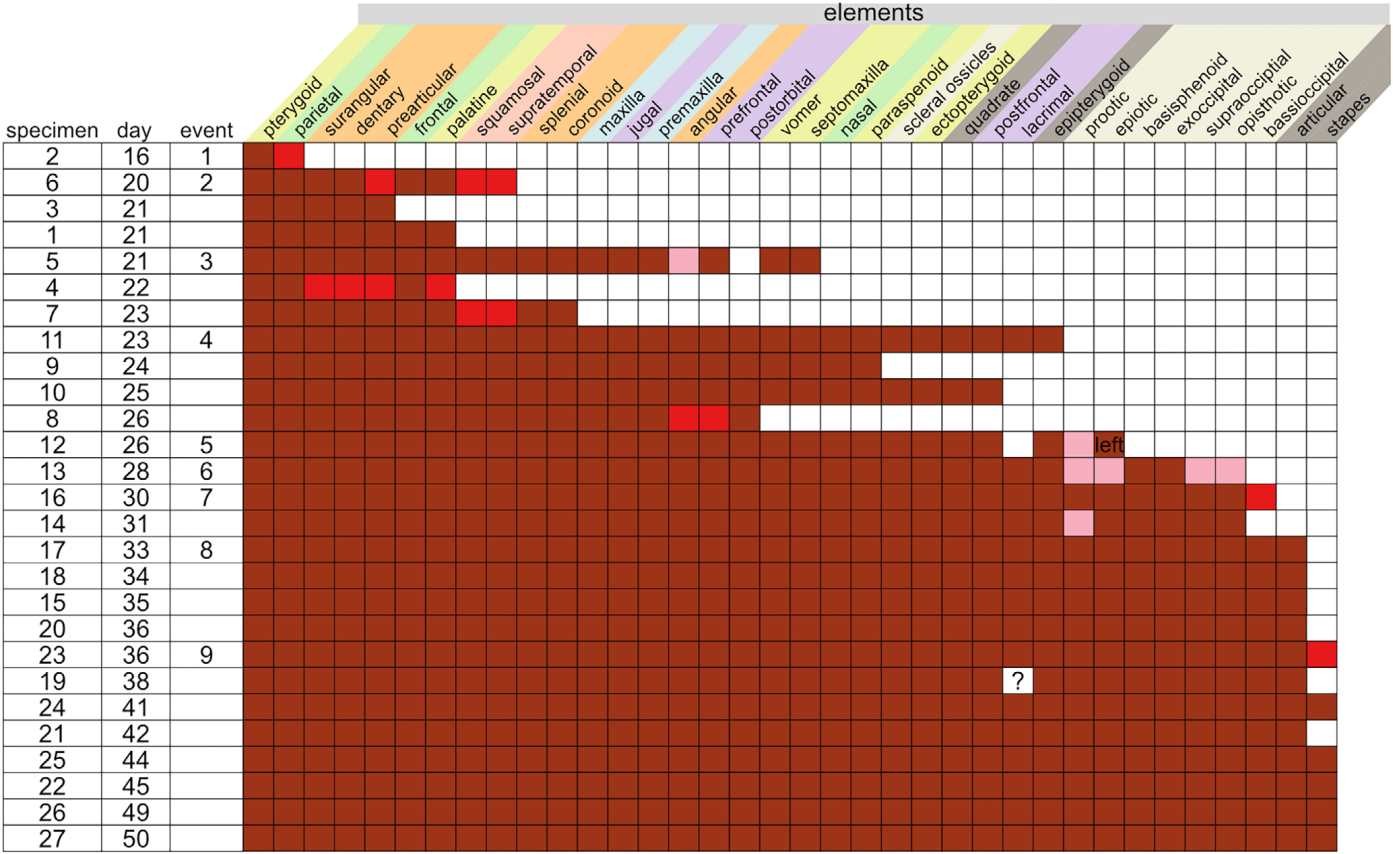
Cranial element mineralization sequence data gleaned from 27 embryos of *Eublepharis macularius* with the specimens (1–27) arranged in days post‐oviposition order (see Table 2). The “event” column identifies the nine mineralization events documented in the text. Filled cells record evidence of mineralization as revealed by staining with Alizarin red, with pink fill indicating weak staining, light red fill indicating moderate staining, and dark red fill indicating strong staining. Cranial entities are listed across the top of the figure, with color‐coded background fill indicating groups of purportedly anatomically associated elements (Evans, 2008; Schoch, 2006), as indicated in Figure 1.

When ordered by day of development, we found the variability in the data to be greater than that revealed by ordering by HL (Figures 1 and 2). The stepped pattern of sequential events (Figure 2) expresses a more jagged and incised right profile than does that for the HL‐based data (Figure 1).

Employing day of development as the chronological timetable results in only nine discrete mineralization events able to be teased out of the data set. These are similar, but not in all cases identical, to the clusters of elements revealed using the HL data. The following elements or clusters of elements appear in sequential order (Figure 2): (1) pterygoid and parietal; (2) surangular, dentary, prearticular, frontal, palatine, squamosal, supratemporal; (3) splenial, coronoid, maxilla, jugal, premaxilla, angular, prefrontal, vomer, septomaxilla; (4) postorbital, nasal, parasphenoid, scleral ossicles, ectopterygoid, quadrate, postfrontal, lacrimal, epipterygoid; (5) prootic, epiotic; (6) basisphenoid, exoccipital, supraoccipital, opisthotic; (7) basioccipital; (8) articular; (9) stapes.

Attempting to further refine the day of development sequence by employing intensity of staining of elements (Figure 2) reveals the following: (a) the pterygoid mineralizes before the parietal; (b) the palatine and frontal mineralize before the prearticular, squamosal, and supratemporal; (c) the splenial, coronoid, maxilla, jugal, premaxilla, prefrontal, vomer, and septomaxilla mineralize before the angular; (d) the epiotic mineralizes before the prootic; and (e) the basisphenoid and exoccipital mineralize before the supraoccipital and opisthotic. This provides a maximum of 14 resolved mineralization events (as opposed to 9 from the data without using staining intensity as a supplementary criterion).

As for the HL data (Figure 1), the density of sampling in relation to the variation evident (Figure 2) has the potential to obscure and obfuscate the signal. By selecting the embryos at 16, 21, 22, 23, 25, 26, 28, 30, 33, and 36 days of development (specimen numbers 2, 3, 4, 7, 10, 12, 13, 16, 17, 23—Figure 2) the combination of day of development and intensity of staining data yields a total of 16 discrete mineralization events. This provides a better level of resolution than that based upon the size‐ordered (HL) specimens or the unfiltered data based upon developmental day (dpo), but is dependent upon the vagaries of sampling.

### 3.3 Resolution of mineralization sequence using developmental stage as the proxy for developmental time

**FIGURE 3:**
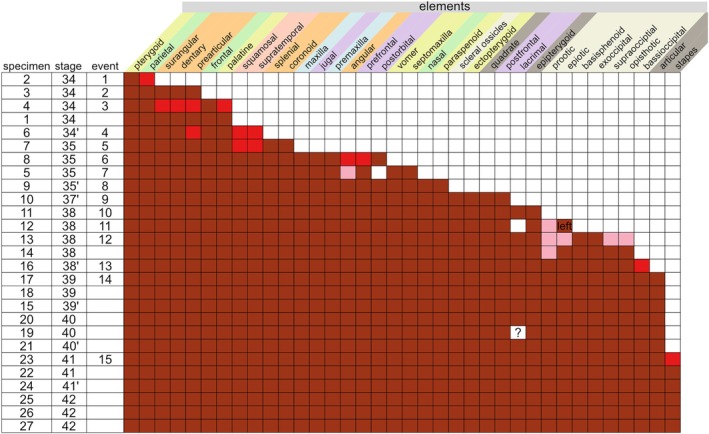
Cranial element mineralization sequence data gleaned from 27 embryos of *Eublepharis macularius* with the specimens (1–27) arranged in developmental stage and substage order (see Table 2). The “event” column identifies the 15 mineralization events documented in the text. Arrangement of individuals within given development stages based upon the increasing number of cranial elements exhibiting evidence of mineralization. Filled cells record evidence of mineralization as revealed by staining with Alizarin red, with pink fill indicating weak staining, light red fill indicating moderate staining, and dark red fill indicating strong staining. Cranial entities are listed across the top of the figure, with color‐coded background fill indicating groups of purportedly anatomically associated elements (Evans, 2008; Schoch, 2006), as indicated in Figure 1.

**FIGURE 4:**
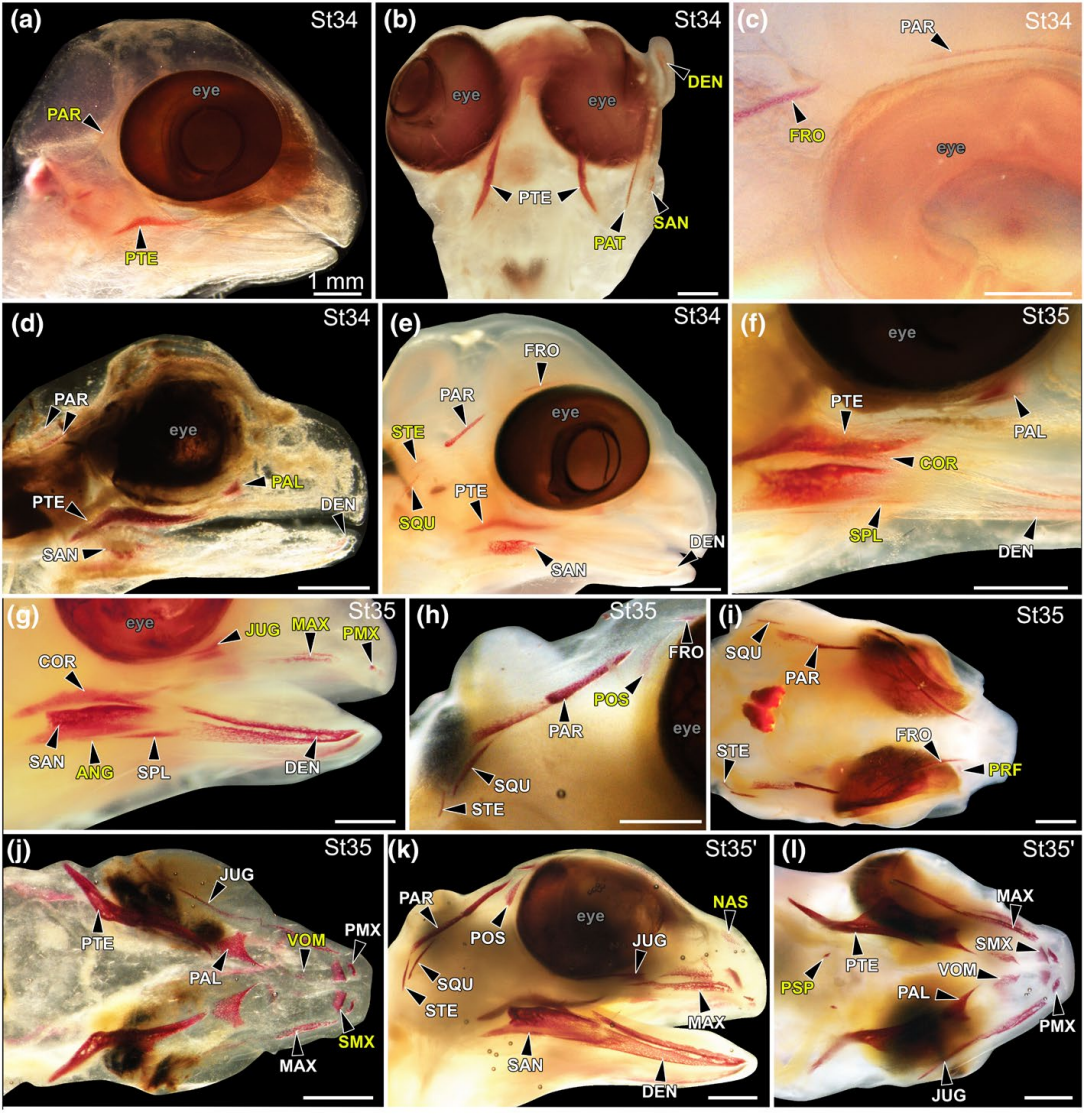
Cranial mineralization sequence events in *Eublepharis macularius* during developmental Stages (St) 34 and 35 (see Figure 3). Abbreviations for each element are presented in yellow on their first appearance and thereafter in white to serve as positional markers. Scale bars = 1 mm. Panels (a, c, d–h, and k) are lateral views; panels (b, i, and l) are dorsal views; panel J is a ventral view. (a) St 34, parietal (PAR), and pterygoid (PTE). (b) St 34, dentary (DEN), prearticular (PAT), and surangular (SAN). (c) St 34, frontal (FRO). (d) St 34, palatine (PAL). (e) St 34, squamosal (SQU), and supratemporal (STE). (f) St 35, coronoid (COR), and splenial (SPL). (g) St 35, angular (ANG), jugal (JUG), maxilla (MAX), and premaxilla (PMX). (h) St 35, postorbital (POS). (i) St 35, prefrontal (PRF). (j) St 35, septomaxilla (SMX), and vomer (VOM). (k) St 35′, nasal (NAS). (l) St 35′, parasphenoid (PSP).

**FIGURE 5:**
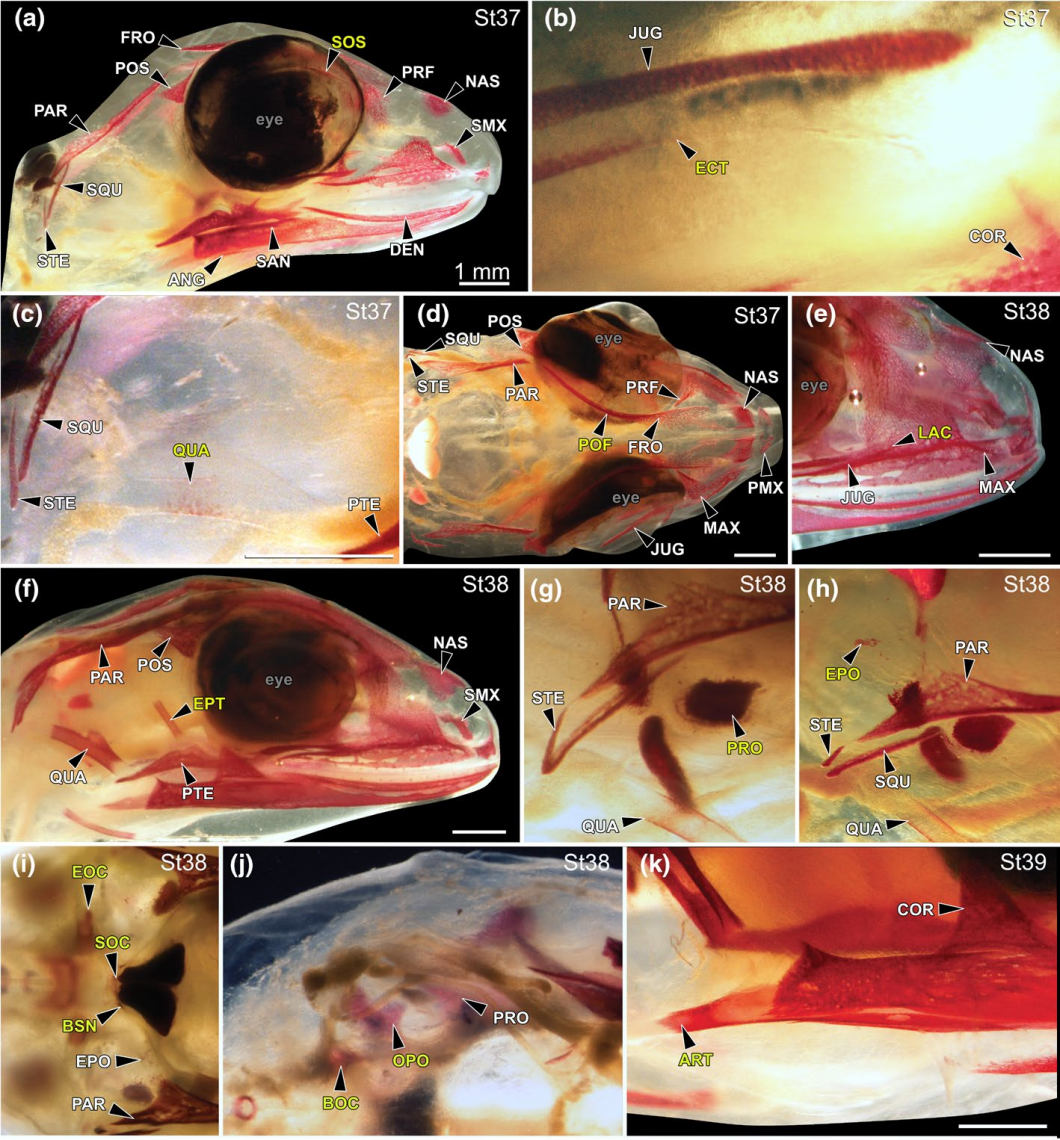
Cranial mineralization sequence events in *Eublepharis macularius* during developmental Stages (St) 37–39 (see Figure 3). Abbreviations for each element are presented in yellow on their first appearance and thereafter in white (see Figure 4 for elements appearing in earlier stages) to serve as positional markers. Scale bars = 1 mm. Panels (a, b, c, f, j, and k) are lateral views; panels (d, h, and i) are dorsal views; panels (e and g) are dorsolateral views. (a) St 37, scleral ossicles (SOS). (b) St 37, ectopterygoid (ECT). (c) St 37, quadrate (QUA). (d) St 37, postfrontal (POF). (e) St 38, lacrimal (LAC). (f) St 38, epipterygoid (EPT). (g) St 38, prootic (PRO). (h) St 38, epiotic (EPO). (i) St 38, basisphenoid (BSN), exoccipital (EOC), and supraoccipital (SOC). (j) St 38, basioccipital (BOC), and opisthotic (OPO). (k) St 39, articular (ART).

Developmental staging has been used as the primary criterion for establishing a chronological scale against which mineralization sequences of lizards are documented (Candan et al., 2024; Hernández‐Jaimes et al., 2012; Jerez et al., 2015; Khannoon & Evans, 2020; Rieppel, 1994; Skawiński et al., 2023; see Table 1). Our data reveal 15 distinct events being resolvable using the developmental stage ordering criterion (Figure 3). The profile of the pattern in Figure 3 is much more regularly stepped than that for the size (HL) (Figure 1) or day of development (Figure 2) data. The following elements or clusters of elements appear in sequential order (Figure 3) as revealed by this criterion for assessment of sequencing: (1) pterygoid, parietal (Figure 4a); (2) surangular, dentary, prearticular (Figure 4b); (3) frontal, palatine (Figure 4c,d); (4) squamosal, supratemporal (Figure 4e); (5) splenial, coronoid (Figure 4f); (6) maxilla, jugal, premaxilla, angular, prefrontal, postorbital (Figure 4g–i); (7) vomer, septomaxilla (Figure 4j); (8) nasal, parasphenoid (Figure 4k,l); (9) scleral ossicles, ectopterygoid, quadrate, postfrontal (Figure 5a–d); (10) lacrimal, epipterygoid (Figure 5e,f); (11) prootic, epiotic (Figure 5g,h); (12) basisphenoid, exoccipital, supraoccipital (Figure 5i,j), opisthotic (Figure 5j); (13) basioccipital (Figure 5j); (14) articular (Figure 5k); and (15) stapes.

Using intensity of staining (Figure 3) as a supplemental indicator of mineralization sequence enables the following to be determined for the staging‐based developmental chronology: (a) the pterygoid mineralizes before the parietal; (b) the frontal mineralizes before the palatine; (c) the jugal, maxilla, postorbital and premaxilla mineralize before the angular and prefrontal; (d) the prootic mineralizes after the epiotic; and (f) the supraoccipital and opisthotic mineralize after the basisphenoid and exoccipital. This brings the total number of mineralization events to 20.

It is evident from the developmental stage data (Figure 3) that nine elements commence mineralization in Stage 34; 12 elements initiate mineralization in Stage35; none in Stage 36; 4 in Stage 37; 9 elements in Stage 38; 1 in Stage 39; none in Stage 40; and 1 in Stage 41. This indicates that the initiation of mineralization of large numbers of elements is closely spaced in time. Classical staging criteria, because individual stages can persist for relatively long periods of developmental time (Table 2—compare column 2, listing days of development, with column 4, documenting developmental stage), may obscure mineralization sequences if they are sampled as singular entities. Our sampling within stages (Figure 3, Table 2) permits resolution within, as well as between, these developmental phases.

### 3.4 Resolution of mineralization sequence using developmental stage and substage as the proxy for developmental time

We subdivided the classical embryological stages (Wise et al., 2009) into smaller subunits, each recognized by morphological changes occurring within the confines of a particular developmental stage (see above). Such an approach has been more informally (in terms of the criteria employed for substage recognition) applied by other authors (Khannoon & Evans, 2020: Table 1; Werneburg et al., 2015).

The application of the substage criterion brings a moderate level of refinement to the discrimination of mineralization events in the head skeleton of *Eublepharis macularius*. The recognition of substages 34′, 35′ and 38′ (specimen numbers 6, 9, and 16 in Figure 3) allows these to be placed chronologically last in their respective developmental stage, providing greater clarification of the relative timing of mineralization initiation within stages. Similarly, the recognition of substage 37′ allowed us to determine that mineralization events confined to stage 37 occurred only in the latter part of the total time in which embryos reside in that stage. Employing substages, however, did not bring about further resolution of pattern.

## 4 DISCUSSION

Our comparison of approaches for resolving the mineralization sequence of the cranial elements of *Eublepharis macularius* reveals that size (HL) (Figure 1) and day of development (Figure 2) exhibit much greater variation than developmental stage (Figure 3) in the apparent sequence of events, thereby resulting in a lower level of discrimination between ostensibly independent mineralization events. This was so even though variation in the relative time of appearance and duration of features employed in the staging of lizard embryos has been documented (Andrews & Greene, 2011). Thus, our working hypothesis that all three approaches will yield the same level of resolution of mineralization events is not supported. All embryos in our sample were raised under identical incubation conditions, revealing that there is considerable variance between individuals in relation to these proxies for developmental time in the early phases of the developmental trajectory.

For the 36 elements under investigation, the ability to resolve only 10 events (or, at best, 14 events using the additional criterion of intensity of staining) indicates that size (HL) is only moderately successful as a proxy for developmental time in relation to the discrimination of mineralization events. Indeed, the relatively dense sampling of the embryos during the critical phase of development for determining the onset of cranial element mineralization, combined with size variance as measured against age (Table 2) actually serves to potentially confound resolution. Employing only 10 of the 27 embryos documented in Figure 1 (specimen numbers 1, 5, 8, 9, 10, 11, 12, 13, 15, 22, with HLs of 7.0, 8.0, 9.0 (×2), 10.8, 11.0, 11.2, 12.6, 13.4, and 17.2 mm, respectively; Figure 1) would also yield 10 discrete clusters of mineralization events. The assumption that an embryo of larger size is older (in terms of day of development) than a smaller one is not absolutely reliable (Table 2). Day of development (Figure 2) resolves the mineralization sequence information less well than that sorted by HL.

Incubation conditions (temperature, humidity) remained constant, so intrinsic factors of the embryos likely account for the size‐related variation (Andrews & Greene, 2011). Clearly, size and absolute or relative age (Table 2) do not show a great degree of concordance.

It is evident that most of the initiations of mineralization occur within a relatively small range of HLs (Figure 1). If sampling was spread equally throughout the HL range in which the complete set of cranial bones initiate mineralization, it is unlikely that the resolution between events would improve. As mentioned above, selective sampling of a subset of the complete series of embryos investigated can actually capture greater resolution of events, but for any sampling regime this would be serendipitous.

It is also evident that HL (a measure of growth) is related to changes in head form (a representation of differential development—Wise et al., 2009: figs. 3–6). Changes in head form, such as relative sizes of anatomical structures and relative proportions of head regions, are likely associated with mineralization events because the elements under consideration do not develop in isolation but instead have intimate relationships with adjacent soft tissues (Cooper et al., 2025; Santos‐Durán et al., 2025). The stage‐correlated mineralization sequence data (Figure 3) indicate that the majority of initiations of mineralization occur in stages 34 to 38. These stages (Wise et al., 2009: figs. 4 and 5) are associated with marked changes in head form. The head in Stage 34 is relatively short (Wise et al., 2009: fig. 4f), with relatively large eyes, short rostrum, and short but tall postorbital region. From Stage 34 to 39 (Figures 4 and 5) the eyes become relatively smaller, the rostrum elongates markedly, and the postorbital region becomes relatively depressed and elongated, with the proportional distance between eye and ear increasing. These changes in form and proportionality of the head are associated with distinctive patterns of the initiation of mineralization of cranial elements, likely accounting for the rather inconsistent signal that emerges when absolute size (HL) is used as a proxy for developmental time. These middle stages of post‐ovipositional development appear morphologically similar in other gekkotan species (Alturk & Khannoon, 2020; Griffing et al., 2019, 2021, 2022, 2024), so we predict that these osteogenic events are similarly sequenced in these. Size (as represented by a linear dimension) and form (the three‐dimensional configuration of the region under consideration) are not necessarily directly related, but the latter is seemingly more evidently related to staging criteria.

The data organized by stage (Figure 3) reveal a pattern with much less variation (variation being a problem for the resolution of mineralization sequences; Hanken & Hall, 1984; Maxwell et al., 2010; Shiel et al., 2014; Spiekman & Werneburg, 2017) than those ordered by size (HL) (relative time) or day of development (real time). Stages have particular real‐time durations (Wise et al., 2009) but these are variable between stages (and probably between individuals), although they represent ordered progressions of developmental events. Thus, the actual duration of real time involved may vary, but it is likely that events within and between stages play out in a consistent fashion.

Overall, the data ordered by developmental stage perform substantially better than when ordered by either size (HL) or day of development in relation to discriminating between initiation of mineralization events. They also provide potential indicators that will assist in sampling. Developmental stages residing in the portion of development in which cranial mineralization initiation begins are few (Table 1). Dividing these into substages, definable by externally visible morphological changes, provides a potential vehicle for isolating portions of development in which apparently simultaneous events occur.

An encouraging aspect of the data arranged according to developmental stage is that selective sampling of subsets of the embryos does not reveal any changes to the resolved sequence, in contrast to when the data are ordered by size (HL) or day of development. Adding more specimens within stages thus has the potential for breaking ties between elements without introducing more variance. Examining the data for the first three Stage 34 embryos (Figure 1, specimens 2, 3, and 4), for example, reveals that three sets of mineralization events can be teased out of the available observations within this stage. Furthermore, staining intensity data allow us to hypothesize that further resolution of mineralization sequencing events can be attained through more nuanced sampling within stages.

Although it is generally advocated that mineralization sequence data provide their own inherent means of standardization (Smith, 2002), these events take place in an integrated developing organism. Understanding not only when mineralization events occur in relation to each other, but why they occur at particular points in developmental time, can only be approached by taking into account other morphological changes that are occurring in concert with the mineralization events.

We advocate, therefore, that wherever possible mineralization sequences be examined by correlating them with staging data (either classical staging subdivisions or SES subdivisions). Staging data more effectively allow critical points along the developmental trajectory to be sampled intensively, permitting determination of whether apparently simultaneous initiations of mineralization can be resolved into more discrete sequences. This may assist in attempting to understand the order displayed by mineralization sequences.

Ideally sampling using either size, day of development or developmental stage to determine the sequence of mineralization would be continuous, but practical limitations necessitate that it will more likely be episodic. For example, using size (HL or some other aspect of length, such as SVL) to assign embryos to size classes has generally been achieved without recourse to standardizing the increments (Good, 1995; Rieppel, 1992), although generally the range of embryo sizes has spanned SVLs over a 2.0‐ to 2.5‐fold increase (1.2–2.6 fold increase for the size ranges for the species included in Table 1). For our data, a HL increase of 3.2‐fold was necessary to capture all the events, and spacing of samples had to be close to accomplish this (Figure 1). This is especially so for the earliest phases of the initiation of mineralization events, with almost all of them taking place in the HL range of 7.0–12.6 mm, encompassing an HL increase of only 5.6 mm. The remaining increase of 9.8 mm in HL captured only two additional events. Likewise, day of development, the least successful of the approaches in terms of event discrimination (Figure 2), incorporated most of the mineralization events (Figure 2, Table 2) in the time span of 16–26 days, with the remaining 24 days capturing only three additional events. It is likely that both such approaches would reveal quite different outcomes for different species, making the selection of an effective sampling regime difficult and imprecise, especially as different size ranges and numbers of developmental days will be involved (Table 1).

Developmental staging, however, presents a different type of problem, resulting from the relatively few developmental stages that are encompassed by the entire pattern of mineralization initiation events. For *Eublepharis macularius* Stage 34 (Figure 3) encompasses five separate mineralization events, Stage 35 incorporates a further three; and Stage 38 four events (Figure 3). In contrast, Stages 36, 37, and 39–41 collectively capture very few events. Thus, employing the staging criterion indicates that events are, for the most part, clustered in a few stages that collectively span a relatively large proportion of the total number of developmental days over which the totality of the initiation of mineralization events occurs (Table 2). Collectively Stages 34 and 35 encompass 13 developmental days, and Stage 38 accounts for 8 developmental days. The length of stages thus also potentially renders their employment problematic for targeting mineralization events because much happens within a relatively few stages. Furthermore, these stages can be somewhat variable in the absolute amount of time involved, depending upon the lizard lineage (Andrews et al., 2013). Identification of substages within developmental stages, using additional externally visible developmental markers, allowed us to break stages into smaller units, potentially permitting the targeting of mineralization events within stages as well as between them. Other authors (Kaczmarek et al., 2020) have identified intermediate conditions within or between “classical” developmental stages, or substages within recognized stages, based upon internal anatomical features of gekkotans. This differs somewhat from our objective, which was to identify developmental progression within recognized developmental stages as markers to permit assessment of internal anatomical developmental events within those stages. Our substage markers (see above) allowed us to place embryos in a developmental sequence prior to the exploration of the mineralization sequence of cranial elements rather than employing the mineralization events to establish substages.

Our sampling of the embryos of *Eublepharis macularius* was quite dense, but we were still only about 55% successful in resolving the sequence of mineralization into a set of unique events (36 elements were at best segregated into 20 events by employing the intensity of staining criterion as well as actual discrete initiations for the developmental stage and substage data; Table 3). This outcome, however, represents a much more resolved pattern of cranial mineralization sequence for geckos than was previously available (Table 3), corroborating our prediction on this count. This outcome also far exceeds the previously best resolution pattern attained for lizards (Table 1; Werneburg et al., 2015 for *Varanus panoptes*), resulting in far fewer clusters of tied initiations. Given the much greater size of the embryos of *V. panoptes* and its much longer *in ovo* development period (Table 1; Werneburg et al., 2015), our ability to more adequately document the cranial mineralization sequence of the comparatively small *E. macularius*, with its relatively short period of *in ovo* development, endorses the employment of dense sampling of developmental stages and substages as a preferred approach to compartmentalizing the events involved.

Given the variation that is evident in the initiation of mineralization between elements (Figure 3), the intensity of the sampling, and the small span of developmental time that is involved, it is likely that further resolution will be difficult to attain even for the best‐resolved scenario. Whether or not simultaneity of initiation of mineralization events is rare in an absolute sense (Bininda‐Emonds et al., 2002; Hugi et al., 2010), it may continue to persist in explorations of mineralization sequences unless the intensity of sampling in the relatively small window of the totality of developmental time in which skeletal mineralization commences is greatly increased.

Our findings indicate that developmental stage as an ordering criterion against which mineralization sequences in lizards can be assembled provides a framework for obtaining the greatest level of discrimination relative to sampling effort and intensity. Size (HL) and day of development, by contrast, are significantly less effective in approaching full resolution. The events in question are compressed into a relatively short period of total *in ovo* developmental time, as is evident from previous studies and the number of ties evident in these accounts (Table 1). If staging (and substaging) can be established for the taxa being investigated, then this offers the greatest promise for targeting periods of intense initiation of mineralization activity because it should be able to be applied uniformly across species (regardless of absolute size of the embryos and differences in absolute lengths of the developmental period).

## AUTHOR CONTRIBUTIONS

Contributions to the conceptualization and design of the study: APR, PADW; Acquisition of data: PADW; Data analysis and interpretation: PADW, APR, AHG; Drafting of the manuscript: APR, PADW; Critical review of the manuscript: APR, AHG, PADW; Approval of the article: PADW, AHG, APR.

## Data Availability

The data that support the findings of this study are available from the corresponding author upon reasonable request.
